# Calcineurin inhibitors cyclosporine A and tacrolimus induce vascular inflammation and endothelial activation through TLR4 signaling

**DOI:** 10.1038/srep27915

**Published:** 2016-06-13

**Authors:** Raquel Rodrigues-Diez, Cristian González-Guerrero, Carlos Ocaña-Salceda, Raúl R. Rodrigues-Diez, Jesús Egido, Alberto Ortiz, Marta Ruiz-Ortega, Adrián M. Ramos

**Affiliations:** 1 Laboratory of Nephrology and Vascular Pathology, Instituto de Investigación Sanitaria-Fundación Jiménez Díaz (IIS-FJD), Madrid, Spain; 2 Cellular Biology in Renal Diseases Laboratory, Universidad Autónoma de Madrid, Instituto de Investigación Sanitaria-Fundación Jiménez Díaz (IIS-FJD), Madrid, Spain; 3 Fundación Renal Íñigo Álvarez de Toledo (FRIAT), Madrid, Spain

## Abstract

The introduction of the calcineurin inhibitors (CNIs) cyclosporine and tacrolimus greatly reduced the rate of allograft rejection, although their chronic use is marred by a range of side effects, among them vascular toxicity. In transplant patients, it is proved that innate immunity promotes vascular injury triggered by ischemia-reperfusion damage, atherosclerosis and hypertension. We hypothesized that activation of the innate immunity and inflammation may contribute to CNI toxicity, therefore we investigated whether TLR4 mediates toxic responses of CNIs in the vasculature. Cyclosporine and tacrolimus increased the production of proinflammatory cytokines and endothelial activation markers in cultured murine endothelial and vascular smooth muscle cells as well as in *ex vivo* cultures of murine aortas. CNI-induced proinflammatory events were prevented by pharmacological inhibition of TLR4. Moreover, CNIs were unable to induce inflammation and endothelial activation in aortas from TLR4^−/−^ mice. CNI-induced cytokine and adhesion molecules synthesis in endothelial cells occurred even in the absence of calcineurin, although its expression was required for maximal effect through upregulation of TLR4 signaling. CNI-induced TLR4 activity increased O_2_^−^/ROS production and NF-κB-regulated synthesis of proinflammatory factors in cultured as well as aortic endothelial and VSMCs. These data provide new insight into the mechanisms associated with CNI vascular inflammation.

Chronic allograft vasculopathy is a pathological condition that impairs endothelial function and integrity and negatively impacts on the half-life of both solid organ engrafted and patients. Among many other causes that contribute to chronic allograft vasculopathy, such as immune rejection, ischemic damage, hypertension and atherosclerosis, it is widely known that therapies based on the use of the calcineurin inhibitors (CNIs) cyclosporine A (CsA) and tacrolimus are main contributors in the development of this transplant-associated complication^1^,^2^,^3^,^4^.

Endothelial damage and dysfunction that results in systemic hypertension are among the most recognized vascular affections in solid organ transplantation patients on CNIs. Mechanistically, apoptosis, oxidative stress and the inhibition of endothelium-derived NO production may contribute to CNI-induced endothelial cell damage and dysfunction^5^,^6^,^7^. Moreover, there are evidences that vascular toxicity of CNIs also implicates vascular smooth muscle cells (VSMCs)^8^. Moreover, higher expression levels of TGFβ-1 receptor in endothelial cells caused renal arteriolar hyalinosis, which is associated to kidney dysfunction and glomerulosclerosis^9^. Even though CNIs potently reduce the activation of the nuclear factor kappa-light-chain-enhancer of activated B cells (NF-κB) in T cells, we have demonstrated the opposite effect in renal tubular cells^10^. Nonetheless, whether a similar NF-κB activation leading to inflammation can occur in vascular cells exposed to CNIs has not been addressed yet.

Activation of the innate immune system resulting in inflammation is an adaptive response aiming at eliminating invading microorganisms or re-establishing normal tissue functions after damage^11^. However, sustained stimulation of the innate immunity may lead to inflammatory diseases. Toll-like receptors (TLRs) are key sensors of the innate immune system which are widely distributed in immune cells as well as in other cell types, including endothelial and VSMCs. Activation of TLRs results from binding to microbial toxins or pathogen-associated molecular patterns (PAMPs) and to intracellular ligands or damaged-associated molecular patterns (DAMPs) released from stressed tissues. Signaling through TLR4 requires engagement of the cytoplasmic adaptor molecules Myeloid differentiation factor 88 (MyD88) and Toll/IL-1 receptor (TIR) domain-containing adaptor protein inducing interferon-*β* (IFN*β*) (TRIF). Main downstream targets of MyD88 and TRIF-adapted signaling are the NF-κB pathway, the MAPK cascades and the interferon pathway which is exclusively regulated from TRIF-derived signals^12^. A common end outcome in cell expressing TLR4 is the synthesis of many proinflammatory molecules, namely cytokines, chemokines and soluble mediators of inflammation^13^. On the other hand, TLR signaling must be restrained to avoid damaging and improper inflammatory responses through a battery of soluble decoy TLRs and intracellular negative regulators for TLR4-derived signaling pathways^14^. TLR4 expressed on endothelial and VSMCs has been proposed to contribute to vascular inflammatory responses^15^,^16^. TLR4 is also required for triggering early vascular events accessory to inflammation, such as endothelial activation during ischemic damage in acute kidney injury and allogenic kidney transplantation^18^,^19^. Moreover, recent investigations have highlighted a role of TLR4 in experimental vascular injury by mediating not only inflammation but also dysfunction, remodelling and stiffness associated with spontaneous or induced hypertension in rats^20^. These data pointed to a role of TLR4 activation in vascular damage and dysfunction. Nonetheless, the involvement of TLR4 as mediator of CNI-induced vascular injury has not been studied to date. We show in this study that in relevant vascular cell types and in aortic tissue, CNIs induce vascular inflammation through a previously non described mechanism involving TLR4 activation.

## Material and Methods

### Cells and reagents

The murine MS1 (Mile Sven 1) pancreatic islet endothelial cell line, obtained from American Type Culture Collection (ATCC®, CRL-2279^TM^), is a commonly used model of microvascular endothelium. MS1 cells were cultured in high glucose (4.5 g/l) Dulbecco’s modified Eagle’s medium (DMEM), supplemented with 5% decomplemented (heat inactivated) fetal bovine serum (FBS), 120 U/mL streptomycin/penicillin and 4 mM L-Glutamine, in 5% CO_2_ at 37 °C. MOVAS (ATCC®, CRL-2797^TM^) is an established VSMC line from C57BL/6 mice. Cells were grown in DMEM supplemented with 10% FBS, 2 mM L-Glutamine and 0.2 mg/ml G-418 (all reagents obtained from Lonza). For experiments, cells at 80% confluence were growth-arrested by serum starvation for 24 h. Endotoxin-free CsA (Calbiochem, Merck Chemicals) and tacrolimus (USBiological) stock solutions (10 mg/ml) were dissolved in ethanol. CLI-095 (InvivoGen) was used according to manufacturer’s time and dose recommendations. The NF-κB inhibitor parthenolide, the TRIF inhibitor resveratrol and the antioxidants 4′-hydroxy-3′methoxyacetophenone (apocynin) and diphenyleneiodonium chloride (DPI) were from Sigma-Aldrich. These reagents were used at concentration derived from prior dose-response studies in our laboratory or from bibliographic data.

### Gene expression studies

One μg RNA isolated by Tripure (Roche) was reverse transcribed with High Capacity cDNA Archive Kit and real-time PCR was performed on a ABI Prism 7500 PCR system (Applied Biosystems, Thermo Fisher Scientific) using the DeltaDelta Ct method. RT-PCR was performed using the following fluorogenic (FAM or VIC) predesigned primers (Applied Biosystems, Thermo Fisher Scientific): Ccl2/MCP1 (Mm00441242_m1), Ccl5/RANTES (Mm01302428_m1), Il6 (Mm00446190_m1), Tnfa (Mm00443258_m1), Icam1 (Mm00516023_m1), Vcam1 (Mm01320970_m1), Sele (Mm01310197_m1), Et1 (Mm00438656_m1), Infb1 (Mm00439552_s1), IRF1 (Mm01288580_m1), IRF7 (Mm00516793_m1). Results were expressed in copy numbers, calculated relative to unstimulated cells or aortas, after normalization against GAPDH using the ABIPrism 7500 Fast sequence detection PCR system software (Applied Biosystems).

### ELISA

Cells were stimulated with CsA (10 μg/ml) or tacrolimus (20 μg/ml) and murine MCP-1 and VCAM-1 were determined in the supernatants by ELISA according to manufacturer’s recommendations (BD).

### Gene silencing

Gene silencing by RNA interference in cultured cells was performed using predesigned small interfering RNA (siRNA) corresponding to MyD88 (#S201719), calcineurin (#S72075) or a scrambled siRNAs (#4390843) (Ambion, Thermo Fisher Scientific). Subconfluent cells were overnight transfected with 25 nM (MyD88), 100 nM (calcineurin) siRNA or with the scrambled siRNA used at the same concentration as the specific siRNA, employing 50 nM Lipofectamine RNAiMAX (Invitrogen) or treated only with lipofectamine vehicle, according to the manufacturer’s instructions. Then, cells were incubated with 10% heat-inactivated (FBS) for 24 h, followed by 24 h in serum-free medium before the experiments.

### Western blot

Protein content from cell extracts homogenized in lysis buffer (50 mmol/L Tris, 150 mmol/L NaCl, 2 mmol/L EDTA, 2 mmol/L EGTA, 0.2% Triton X-100, 0.3% NP-40, 0.1 mmol/L PMSF, 25 mmol/L NaF) was determined by the bicinchoninic acid method (Pierce Biotechnology). Proteins were separated by 10% SDS-PAGE under reducing conditions and then blotted onto nitrocellulose membranes. Membranes were blocked with 5% skim milk in TBS-T (0.05 mol/L Tris, 0.15 mol/L NaCl, 0.05% Tween 20, pH 7.4). Thereafter, membranes were overnight probed at 4 °C with specific primary antibodies in the same blocking solution or 5% BSA in TBS-T and then incubated with secondary HRP-conjugated antibodies for 1 h at room temperature. Primary antibodies used were: MyD88 (#4283), phospho-IκBα (#2859) and phospho-p65 (Ser536) (#3033) from Cell Signaling Technology; calcineurin (panA) (#07-1491) from Merck Millipore and phospho-IRF3 (pSer936) (#SAB4504031) from Sigma-Aldrich. Antibodies to α-Tubulin (Sigma-Aldrich) and mouse polyclonal anti-GAPDH (Merck-Millipore) were used to correct minor differences in protein loading.

### Immunofluorescence

MS1 cells seeded in 24-well culture plates over glass coverslips. Then, cells were fixed in 4% paraformaldehyde/PBS, permeabilized in 0.1% Triton X-100/ BSA 2%/ PBS for 5 minutes, washed with PBS and then blocked with 2% BSA/PBS for 1 h. Then, cells were incubated with rabbit polyclonal anti-RelA/p65 (1:100) (Santa Cruz Biotechnology #sc-372) followed byAlexaFluor^TM^488 conjugated anti-rabbit antibody (1:500, Invitrogen) and nuclei counterstained with DAPI.

Tissue immunofluorescence was carried out in 5 μm thick Tissue Tek OCT-embedded (Sakura Fineted Europe BV) aorta sections. Slides were rehydrated with 2% BSA in PBS during 1 h. After a blocking step, tissue sections were overnight incubated with the following primaries antibodies: anti NF-κB/p65 (1:50) (Santa Cruz Biotechnology #sc-372), anti CD31 (1:50) (Santa Cruz Biotechnology #sc-1506) and anti αSMA-FITC (1:300) (Sigma-Aldrich #F3777). Tissue incubation with p65 plus CD31 or αSMA antibodies was simultaneously carried out. After primary antibody step, tissue samples were incubated with the following secondary antibodies: AlexaFluor^TM^568 conjugated goat anti-rabbit antibody for detection of p65 in endothelial cells (1:250, Invitrogen), AlexaFluor^TM^647 conjugated goat anti-rabbit antibody for detection of p65 in VSMC (1:250, Invitrogen) and AlexaFluor^TM^488 donkey anti goat for CD31 (1:250, Invitrogen). Nuclei were counterstained with DAPI (Sigma-Aldrich).

*In situ* production of O_2_^−^/ROS was evaluated through the oxidative fluorescent dye dihydroethidium (DHE) (Molecular Probes, Thermo Fisher Scientific). Briefly, OCT-embedded aortic section were equilibrated in KHS (30 min, 37 °C) and incubated with DHE (5 μM, 30 min, 37 °C). DHE was detected by excitation at 540 nm and emission at 610 nm. The elastin layer was captured by autofluorescence (excitation at 488 nm). Both cells and tissue section were mounted in ProLong® Gold Antifade Reagent (Invitrogen) and analyzed with a TCS SP5 fluorescent laser scanning confocal microscope (Leica).

### Tissue preparation

Studies were performed in 16–24 weeks old wild-type C57BL/6 mice (Charles River Laboratories) or in TLR4 knockout mice of the same background (kindly provided by Dr. Consuelo Guerri, Centro de Investigación Príncipe Felipe, Spain and originally donated by Dr. S. Akira, Osaka University, Japan). Animals were maintained at the local animal facilities, with free access to food and water, normal light-dark cycles and under special pathogen-free conditions. Mice were sacrificed under anesthesia with Isofluorane (Abbott Laboratories) and aortas were dissected free of fat and connective tissue. Then, tissue sections were placed in culture plates, covered with DMEM medium and left untreated overnight at 37 °C to recover the basal state. Next, aortic segments were stimulated with CsA or tacrolimus alone or in the presence of CLI-095, and then processed according to the procedure of interest. For assessment of O_2_^−^/ROS production and NF-κB/p65 location by confocal microscopy, tissue samples were placed in cold Krebs-Henseleit solution (KHS in mM: 115 NaCl, 25 NaHCO_3_, 4.7 KCl, 1.2 MgSO_4_.7H_2_O, 2.5 CaCl_2_, 1.2 KH_2_PO_4_, 11.1 glucose, and 0.01 Na_2_EDTA) containing 30% sucrose for 20 min, then transferred to a cryomold containing a Tissue Tek OCT-embedding medium (Sakura Finetek Europe BV) and then frozen at −80 °C. For gene expression studies, aorta segments were immediately frozen in liquid nitrogen and kept at −80 °C. All the procedures on animals were performed according to the European Community and Animal Research Ethical Committee guidelines. The animal protocols were approved by the Instituto de Investigación Sanitaria Fundación Jiménez Díaz Animal Research Ethical Committee (body authorized by the Dirección General de Medioambiente, Consejería de medioambiente y Ordenación del Territorio, Comunidad de Madrid, RD 53/2013).

### Statistics

Statistical analysis was performed using SPSS 11.0 (SPSS, Chicago, IL). Results are expressed as mean ± SEM. Significance at the p < 0.05 level was assessed by non-parametric Mann-Whitney test for two independent samples.

## Results

### In murine endothelial cells, CNIs promote the synthesis of NF-κB-dependent chemokines and adhesion proteins

**Figure 1:**
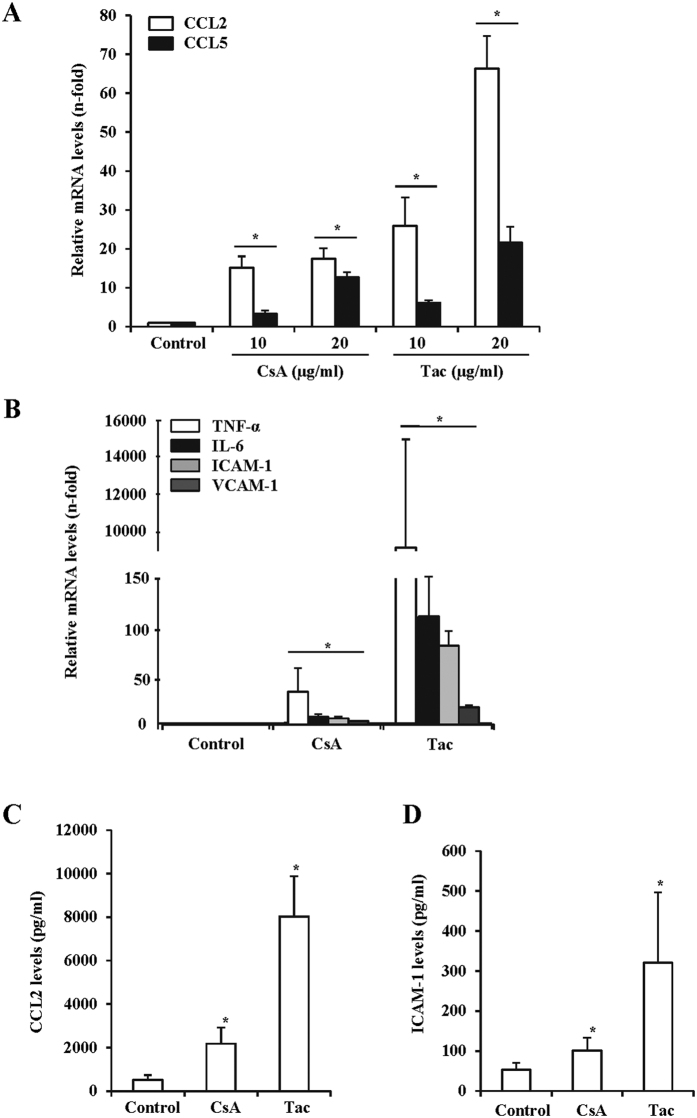
CNIs induce the expression of inflammatory mediators and endothelial activation markers in murine endothelial cells. The expression of proinflammatory cytokines and endothelial activation markers was evaluated in murine endothelial cells exposed to cyclosporine (CsA) or tacrolimus (Tac). (**A)** Dose-dependent CCL2 and CCL5 mRNA expression in cells treated for 6 h with 10–20 μg/ml CsA or Tac, assessed by qRT-PCR. (**B)** TNF-α, IL-6, ICAM-1 and VCAM-1 mRNA expression in cells treated with 10 μg/ml CsA and 20 μg/ml Tac, qRT-PCR. (**C,D)** CCL2 (**C**) or ICAM-1 (**D**) protein levels assessed by ELISA in supernatants from cells stimulated with 10 μg/ml CsA, 20 μg/ml Tac or vehicle (Control). Data represent the mean ± SEM of three independent experiments. *p ≤ 0.05 vs control.

Endothelia are highly exposed to the toxic action of circulating CNIs. To assess whether CNIs induce direct proinflammatory effects in endothelial cells, cultured murine endothelial cells were treated with CsA or tacrolimus under standard *in vitro* dose and time conditions^10^,^21^ and the mRNA and protein expression of key proinflammatory cytokines and adhesion molecules were assessed by PCR and ELISA assays, respectively. Treatment with CsA and tacrolimus caused a dose-dependent upregulation of the chemokines monocyte chemotactic protein-1/chemokine (C-C motif) ligand 2 (MCP1/CCL2) and regulated on activation normal T cell expressed and secreted/chemokine (C-C motif) ligand 5 (RANTES/CCL5) gene expression at 6 h (Fig. 1A). At the same time, CsA and tacrolimus also induced the mRNA synthesis of relevant vascular proinflammatory cytokines and endothelial activation markers, namely IL-6 and TNF-α, and intercellular adhesion molecule 1 (ICAM-1) and vascular cell adhesion molecule 1 (VCAM-1), respectively (Fig. 1B). Accordingly, when supernatants of cultured cells were analyzed by ELISA, a time-dependent accumulation of the corresponding CCL2 and ICAM-1 secreted proteins was also found (Fig. 1C,D).

**Figure 2:**
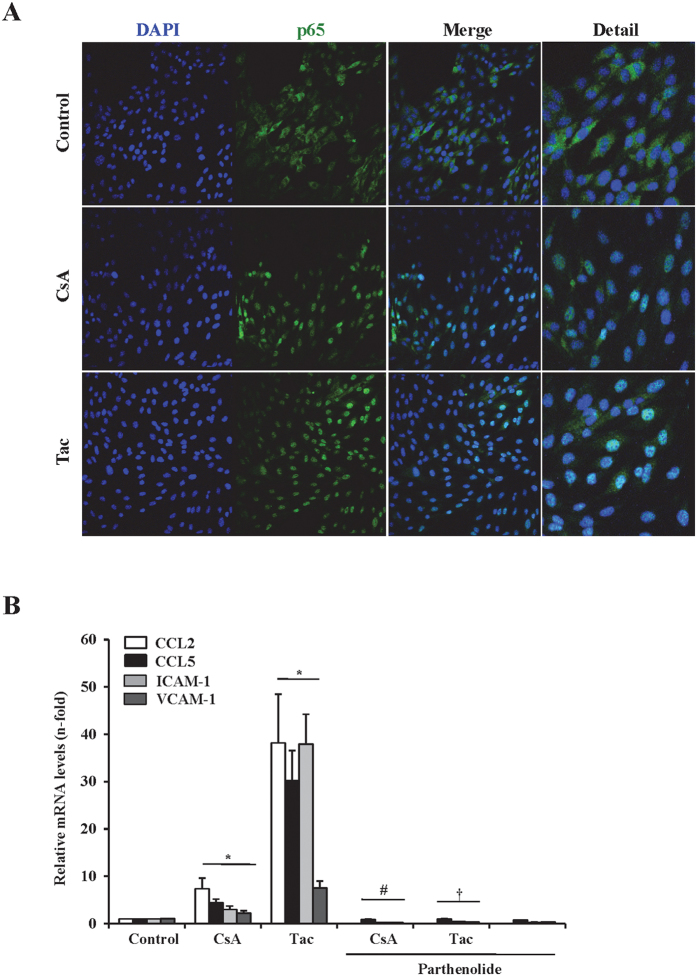
Proinflammatory responses elicited by CNIs in endothelial cells depend on NF-κB activation. (**A)** Representative confocal microscopy immunofluorescence images showing NF-κB/p65 (green) nuclear translocation in cells stimulated for 30 min with 10 μg/ml CsA and 20 μg/ml Tac. Nuclei were counterstained with or with 4′,6-diamino-2-fenilindol (DAPI) (blue). Original magnification x400. **(B)** Addition of the NF-κB inhibitor parthenolide 1 h before stimuli, abolished the CsA or Tac-induced CCL2, CCL5, ICAM-1 and VCAM-1 mRNA upregulation at 6 h. Mean ± SEM of three independent experiments. *p ≤ 0.05 vs control; ^#^p ≤ 0.05 vs CsA, ^†^p ≤ 0.05 vs Tac.

Activation of NF-κB is intrinsically involved in regulating the inflammatory response in the cardiovascular system^22^,^23^. Nuclear translocation of NF-κB/p65 subunit is a key event in NF-κB activation. An increased nuclear NF-κB/p65 content in cells treated with CsA or tacrolimus for 30 min, which is compatible with the upregulated synthesis of proinflammatory factors previously found, was observed by confocal immunofluorescence (Fig. 2A). Indeed, pretreatment of cells with the NFκB inhibitor parthenolide^23^, prevented the CNI-induced CCL2, CCL5, ICAM-1 and VCAM-1 expression (Fig. 2B). Taken together, these results are consistent with a role of NF-κB regulating the CNI-induced proinflammatory events in endothelial cells.

### Toll-like receptor signaling is a key event mediating CNI-induced proinflammatory activity in endothelial cells

**Figure 3:**
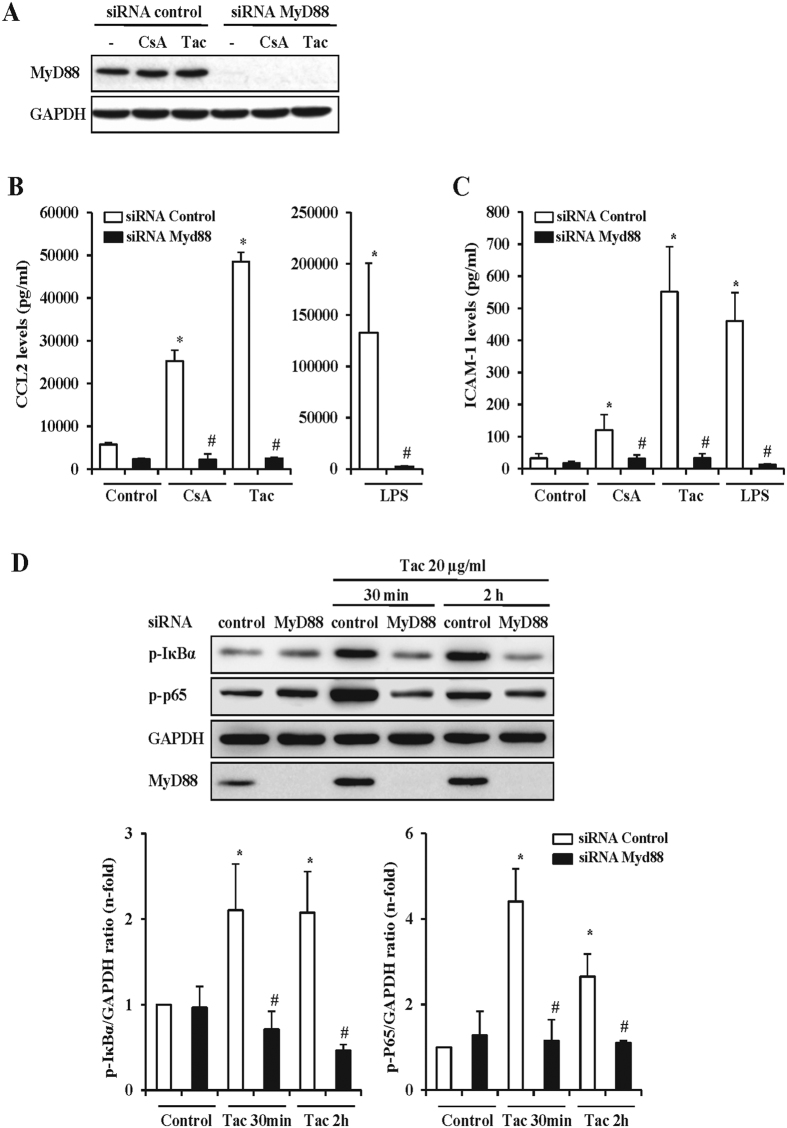
CNI-induced proinflammatory responses in endothelial cells depend on MyD88 signaling. (**A)** The overall efficiency of the transfection procedure was assessed by detection of MyD88 expression by western blot in total protein extracts from cells transfected with a scramble siRNA (sc-siRNA) or with a MyD88 siRNA (MyD88-siRNA). After transfection, cells were stimulated with vehicle, CsA or Tac for 24 h. The figure is a representative experiment showing an almost undetectable expression of MyD88 in MyD88-siRNA transfected cells compared to expression levels in cells transfected with the sc-siRNA. CsA or Tac did not significantly change MyD88 expression. β-actin was used as protein loading control. (**B,C)** Secretion of CCL2 and ICAM-1 was evaluated by ELISA in supernatants from control cells or from sc-siRNA or MyD88-siRNA transfected cells stimulated with CsA, Tac or vehicle for 24 h. Functional efficiency of MyD88 silencing was assessed in cells stimulated for 24 h with 1 μg/ml LPS, used as a control. Bar graphs represent the Mean ± SEM of a set of six independent experiments. (**D)** Activation of the MyD88-dependent NF-κB pathway assessed by phosphorylated IκBα (p-IκBα) and RelA/p65 (p-p65) levels in total protein extracts from sc-siRNA or MyD88-siRNA MS1 transfected cells treated with 20 μg/ml Tac. Image shows representative western blots of p-IκBα and p-p65 from sets of four independent experiments for each protein and the corresponding quantification bar graphs depict the mean ± SEM. A control western blot showing the lack of MyD88 expression in this experiment is also showed at the figure bottom. GAPDH was used as protein loading control. *p < 0.05 vs control (siRNA control); ^#^p < 0.05 vs CsA or Tac (siRNA-MyD88).

The vasculature may react against injury by activating TLRs, which are key innate immunity sensors that promote endothelial activation and inflammation. Since lack of the adaptor protein MyD88 precludes TLR-derived signaling leading to NF-κB activation, we studied the role of TLRs in CNI-induced proinflammatory events by gene silencing of MyD88 expression in endothelial cells. Knockdown of MyD88 was successfully achieved in cells transfected with a specific MyD88-siRNA as judged by the very low protein expression compared to cells transfected with a scramble siRNA (Fig. 3A). In addition, functional assays showed that MyD88 silencing prevented the CCL2 and ICAM-1 synthesis induced by LPS, a known agonist of the TLR/MyD88 pathway (Fig. 3B,C). Significantly, MyD88 knockdown also decreased CNI-induced CCL2 and ICAM-1 protein synthesis, as assessed by ELISA in culture supernatants (Fig. 3B,C). Moreover, taking tacrolimus as representative CNI, MyD88 deficiency resulted in the inhibition of key events leading to NF-κB pathway activation, namely IκBα and p65 phosphorylation (Fig. 3D). These results support the conclusion that engagement of TLR is an initial event that promotes NF-κB activation and hence the synthesis of proinflammatory genes in endothelial cells exposed to CsA or tacrolimus.

### TLR4 is a specific target of CNIs in endothelial cells

**Figure 4:**
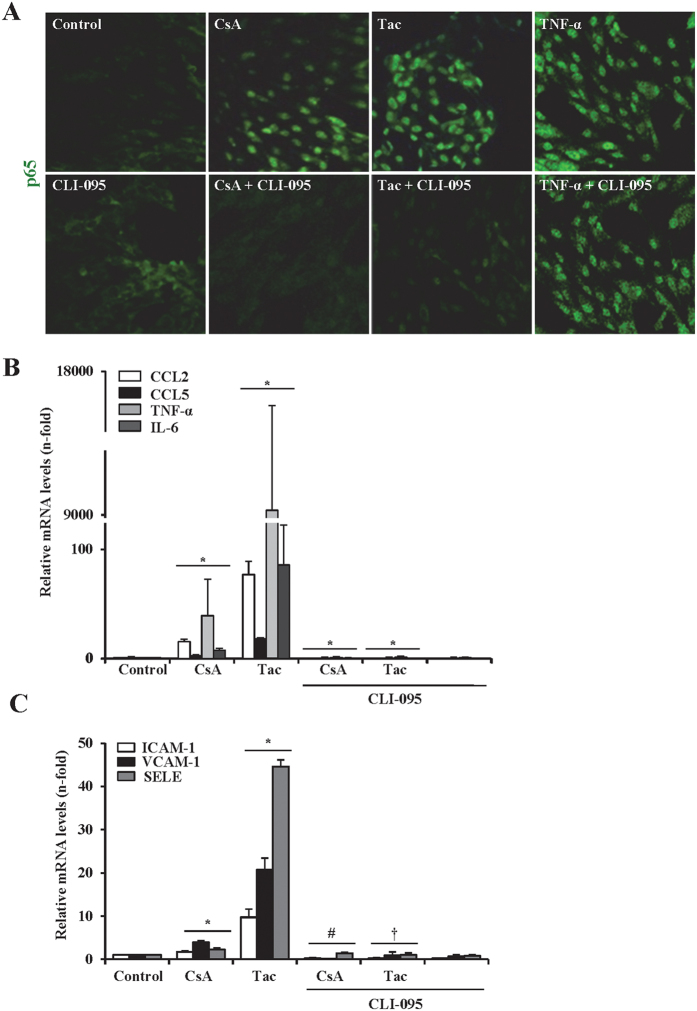
Pharmacological inhibition of TLR4 blocked proinflammatory responses induced by CsA and tacrolimus in endothelial cells. Cells were incubated with 10 μg/ml CsA or 20 μg/ml Tac alone or in the presence of the TLR4 inhibitor, CLI095 (added 6 h before the CNIs). (**A)** Activation of NF-κB was assessed through the nuclear translocation of the NF-κB/p65 subunit detected by immunofluorescence confocal microscopy. Control cells show a cytoplasmic NF-κB/p65 staining (green) whereas in cells stimulated with CsA or Tac, NF-κB/p65 was mostly located inside nuclei. By contrast, the TLR4 inhibitor CLI-095 prevented nuclear translocation of NF-κB/p65. Cells were also stimulated with TNF-α (30 ng/ml) alone as a positive control of NF-κB activation or preincubated with CLI-095 to prove that TLR4 inhibition does not interfere with TNF-α-mediated NF-κB/p65 translocation. (**B,C)** Transcriptional levels of proinflammatory cytokines (CCL2, CCL5, TNF-α, IL-6) (**A**) and endothelial activation markers (ICAM-1, VCAM1, SELE) (**B**) were evaluated by qRT-PCR. Data are mean ± SEM of three independent experiments. *p ≤ 0.05 vs control; ^#^p ≤ 0.05 and ^†^p ≤ 0.05 vs CsA or Tac, respectively.

Based on results obtained in endothelial cells with silenced MyD88, we searched for specific TLRs that could mediate the proinflammatory effects of CNIs. We focused on TLR4 because of its inherent relevance in vascular disease^24^. Pharmacological inhibition of TLR4 with CLI-095, which specifically blocks signaling from the TLR4 intracellular domain, thoroughly repressed CsA- and tacrolimus-induced NF-κB/p65 nuclear translocation (Fig. 4A) and gene expression of inflammatory cytokines represented by CCL2, CCL5, TNF-α and IL-6 (Fig. 4B) and adhesion molecules, including VCAM-1, ICAM-1 and SELE (Fig. 4C). These results indicate a key role of TLR4 promoting CNI-induce endothelial inflammation, since this response was almost completely inhibited by the receptor blockade.

**Figure 5:**
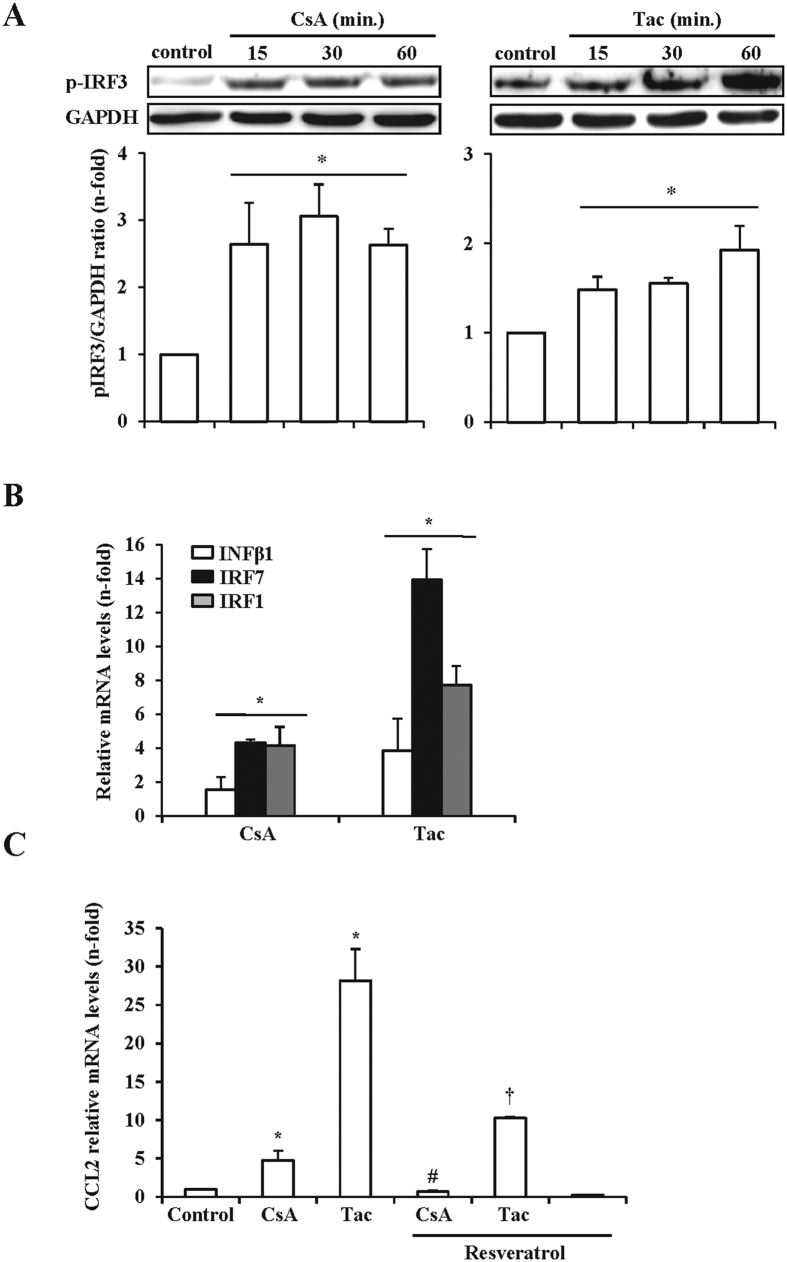
CsA and tacrolimus activate the TLR4/TRIF-dependent signaling pathway in endothelial cells. Cells were treated with CsA or Tac as in the preceding figures. (**A)** Representative western blots of the phosphorylated IRF3 (p-IRF3) using total protein extracts from cells treated with CsA or Tac for 15 to 60 min. Bar graphs represent the mean ± SEM from three independent experiments. *p ≤ 0.05 vs control. (**B)** Activity of the TRIF pathway was assessed by qRT-PCR quantification of IRF3 target gene (INFβ1, IRF7, IRF1) mRNA in cells stimulated with CsA or Tac for 6 h. Data expressed as mean ± SEM of fold-change over control of at least three independent experiments. *p ≤ 0.05 vs control. **(C)** TRIF pathway and proinflammatory response. Cells were stimulated with CsA or Tac for 6 h following pretreatment with 50 μM resveratrol. CCL2 mRNA expression was assessed by qRT-PCR. Bar graph represents mean ± SEM from three independent experiments. *p ≤ 0.05 vs control; ^#^p ≤ 0.05 vs CsA, ^†^p ≤ 0.05 vs Tac.

The preceding results show that in endothelial cells, CNIs activate TLR4 to transduce input signals into MyD88-dependent proinflammatory effects. To study whether CNI also activate a MyD88-independent pathway, we tested for phosphorylation of the transcription factor IRF3, which is activated following TLR4/TRIF engagement, in endothelial cells exposed to CsA or tacrolimus. Immunoblot assays disclosed a rapid increase of IRF3 phosphorylation from 15 minutes after addition of CsA or tacrolimus (Fig. 5A). Moreover, CsA and tacrolimus also activated the transcription of the IRF3 target INFβ1, and also of the INFβ1-regulated proteins IRF1 and IRF7 (Fig. 5B). Thus, these results support the hypothesis that CNIs also trigger the MyD88-independent pathway by recruiting IRF3 at proximal TRIF-adapted TLR4 signaling complexes. Finally, we studied whether TRIF-dependent signaling influenced MyD88-dependent responses. To this end, before incubation with CNIs, endothelial cells were treated with Resveratrol to inhibit the assembly of TBK1 and RIP1 kinases in TLR4-TRIF complexes and hence avoid IRF3 activation^25^. Resveratrol significantly reduced CCL2 gene expression, thus supporting that the TRIF-dependent TLR4 pathway also contributes to the overall CNI-dependent proinflammatory response triggered by TLR4 engagement in endothelial cells (Fig. 5C).

### Calcineurin knockdown partially inhibits the activation of TLR4-dependent pathway elicited by CNIs in endothelial cells

**Figure 6:**
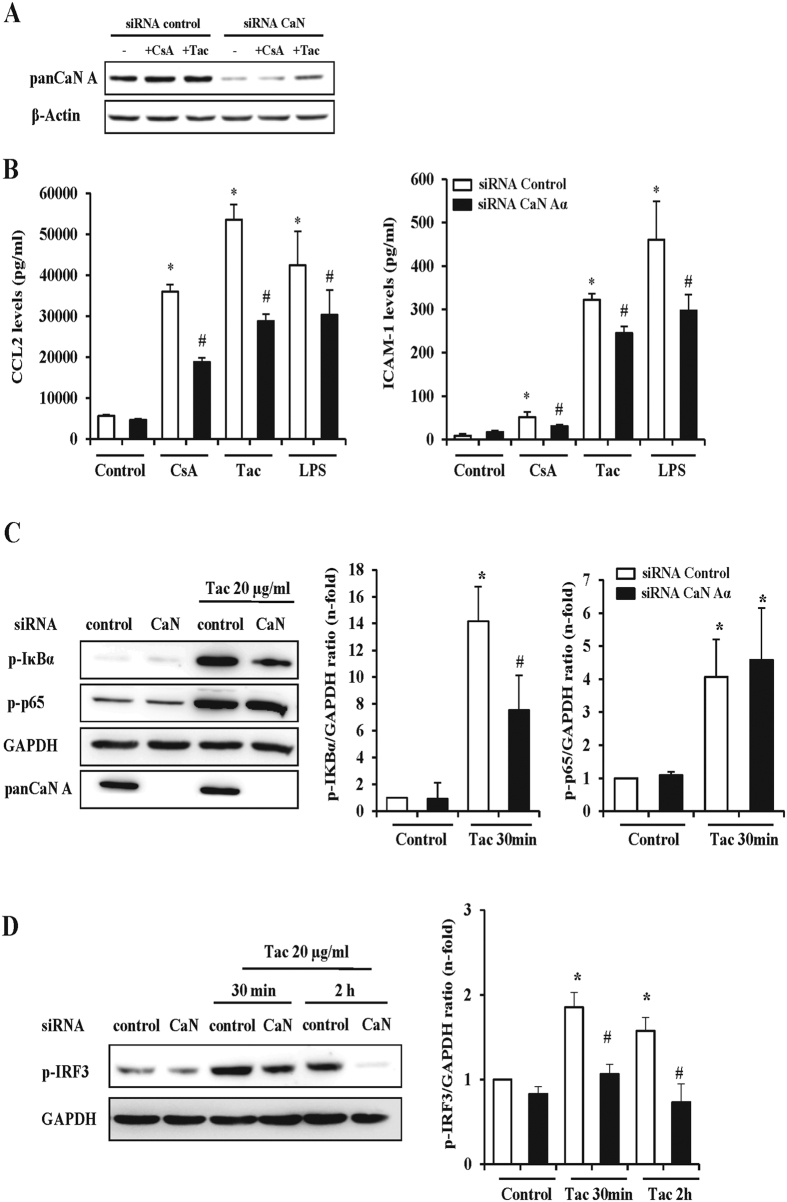
CNI proinflammatory activity in endothelial cells is partially dependent on calcineurin. Cells were transfected with a control scrambled siRNA (sc-RNA) of with a specific calcineurin siRNA (CaN-siRNA) and then stimulated with CsA or Tac to assess the efficiency of the silencing procedure (**A**), the magnitude of the overall proinflammatory response (**B**) or the activation of NF-κB signaling (**C**). (**A)** Representative western blot showing calcineurin expression in control sc-siRNA or in CaN-siRNA cells stimulated with vehicle, CsA or Tac for 24 h. β-actin was used as protein loading control. (**B)** CCL2 and ICAM-1 ELISA in supernatants of sc-siRNA or CaN-siRNA transfected cells stimulated with CsA or Tac for 24 h. As positive control for TLR4 signaling cells were also stimulated with 1 μg/ml LPS. Quantification graphs represent the mean ± SEM of six independent experiments. *p < 0.05 vs sc-siRNA; #p < 0.05 vs Calcineurin-siRNA. (**C)** Comparative levels of calcineurin, phosphorylated IκBα (p-IκBα) and NF-κB/p65 (p-p65) in control, sc-siRNA or in CaN-siRNA transfected cells stimulated with Tac for 24 h. Image shows representative western blots from three independent experiments and the corresponding quantification bar graph. *p < 0.05 vs control (siRNA control); ^#^p < 0.05 vs CsA or Tac (siRNA-Calcineurin). GAPDH was used as protein loading control.

The role of calcineurin in CNI-induced proinflammatory endothelial events was specifically addressed by gene silencing of the α isoform of the catalytic subunit, thus preventing calcineurin activation and calcineurin stabilization by impeding the interaction of CNI-cyclophilin complexes with the regulatory calcineurin B subunit^26^,^27^. In comparison with control cells transfected with the scramble siRNA, calcineurin expression was significantly reduced in endothelial cells transfected with a specific calcineurin siRNA (Fig. 6A). Calcineurin knockdown did not modify CCL2 and ICAM1 secretion by itself but partially inhibited the CNI-induced CCL2 and ICAM1 secretion into the cell culture media (Fig. 6B). To test whether TLR4 signaling modulation by calcineurin is a general mechanism, we stimulated the cells with the specific TLR4 ligand LPS. Accordingly, a quantitatively similar inhibition of CCL2 and ICAM1 production was observed in LPS-treated calcineurin silenced endothelial cells (Fig. 6B). Consistent with the partial reduction of CNI-induced CCL2 and ICAM1 secretion, calcineurin silencing barely downregulated IκBα activation and furthermore, it did not modify p65 phosphorylation, an important event to enhance inflammatory gene transcription, in response to tacrolimus as representative CNI (Fig. 6C). However, activation of the TRIF-dependent pathway induced by CsA and tacrolimus, assessed by detection of phosphorylated IRF3 by western blot, was completely prevented by calcineurin deficiency (Fig. 6D). Overall, these results show that calcineurin absence can downregulate, at least in part, TLR4-derived proinflammatory signals evoked by the CNIs. Thus, normal calcineurin expression could heighten this response.

### CNIs induce proinflammatory cytokine production and endothelial activation in isolated mice aortas and VSMCs

**Figure 7:**
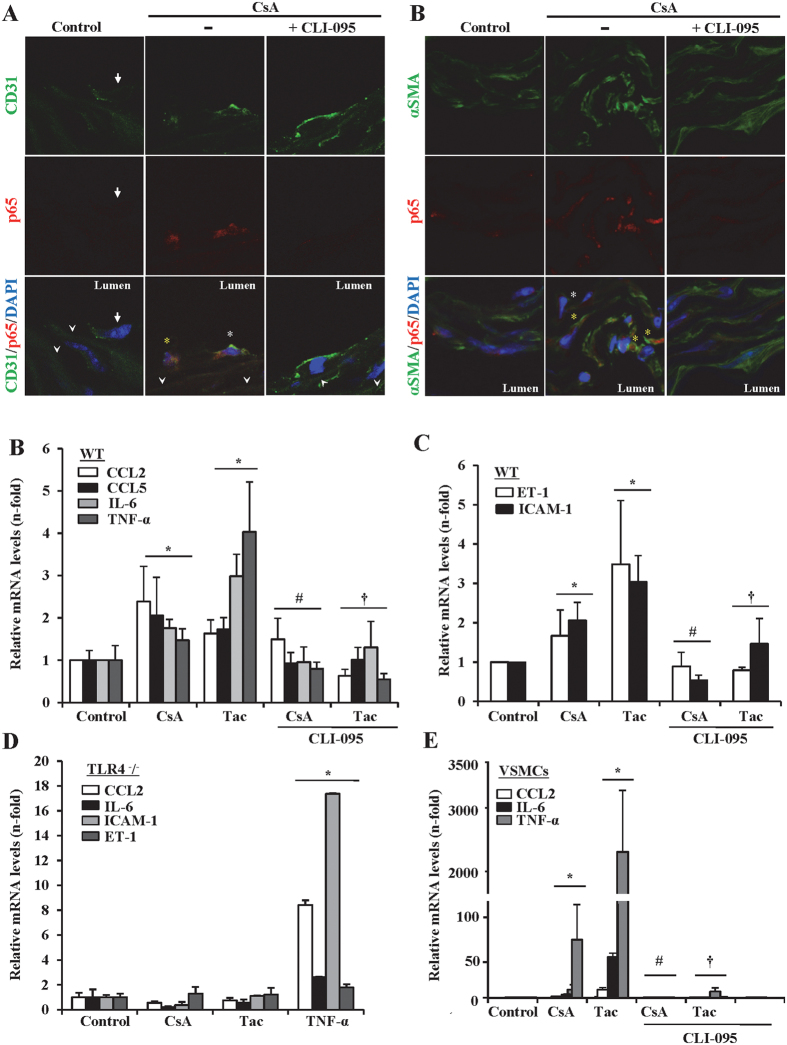
CNI induce inflammation in wild-type aortas but not in aortas from TLR4^−/−^ mice. Aorta tissue segments extracted from wild-type or TLR4^−/−^ C57BL/6 mice were stimulated for 6 h with 10 μg/ml CsA or 20 μg/ml Tac. CLI-095 was added 6 h before stimulation with the CNIs. (**A,B)** Confocal microphotographs showing NF-κB/p65 content and location in control or CsA or CsA plus CLI-095 treated aortic sections from wild-type mice. Activation of NF-κB/p65 was detected by intensification of the specific red fluorescence in cytoplasm and nucleus of either endothelial cells recognized by CD31 staining (green fluorescence surrounding the cell borders) (**A**) or VSMC cells expressing αSMA (cytoplasmic green fluorescence) (**B**). Endothelial cells were found lining the intima layer and facing the lumen and VSMC located in the media layer. Yellow asterisks point cells with nuclear translocation of p65 and white asterisks indicate cells with increased cytoplasmic p65 expression. White arrows point endothelial and VSMC without increased expression of p65 in control or CLI095 treated aortas. White arrowheads in A show elastin fibers (green autofluorescence) which otherwise are not apparently visualized in B because the much higher αSMA specific fluorescence. Nuclei were counterstained with DAPI. Original magnification x630. (**C,D)** Gene expression of CCL2, CCL5, IL-6, TNF-α (left panel) and ICAM-1, ET-1(right panel) in cultured aorta sections from wild-type mice exposed to CsA or Tac alone or in the presence of CLI-095. Data are expressed as mean ± SEM of 4 samples. *p < 0.05 versus control non-stimulated aortas; #p < 0.05 vs CsA or Tac treated aortas. (**E)** Gene expression of CCL2, IL-6, ICAM-1 and ET-1 in cultured aorta sections from TLR4^−/−^ mice exposed to CsA, Tac or TNF-α. Data are the mean ± SEM of 4 samples. *p < 0.05 versus control non-stimulated aortas. (**F)** CCL2, IL-6 and TNF-α mRNA expression in VSMC stimulated for 6 h with 10 μg/ml CsA or 20 μg/ml Tac alone or pretreated with vehicle or CLI-095. Data represent the mean ± SEM of at least three independent experiments. *p ≤ 0.05 vs control; ^#^p ≤ 0.05 and ^†^p ≤ 0.05 vs CsA or Tac alone, respectively.

CNIs alter vascular homeostasis by causing endothelial dysfunction, increased extracellular matrix deposition and hypertension. Subtle endothelial inflammation may contribute to these vascular wall pathogenic processes; however, a proinflammatory effect of CNIs on vascular tissue has not yet been explored. In *ex vivo* aorta cultures, CsA activated NF-κB as indicated by the increased expression and nuclear translocation of the NF-κB/p65 subunit in both endothelial cells (Fig. 7A) and VSMC (Fig. 7B). Similar behavior of p65 was also observed when aortas were treated with tacrolimus (data not shown). Likewise, CsA and tacrolimus also increased mRNA levels encoding NF-κB-regulated proinflammatory cytokines involved in vascular injury, including CCL2, CCL5, IL-6 and TNF-α (Fig. 7C). Moreover, CsA and tacrolimus also increased the mRNA expression of the endothelial activation marker ICAM-1 and the vascular dysfunction marker endothelin-1 (ET-1) (Fig. 7D). To explore whether CNI-induced proinflammatory effects on vascular tissue require TLR4 activation, a pharmacological approach was used. Pretreatment of mice aortas with CLI-095 significantly prevented both NF-κB activation (Fig. 7A,B) and hence the increased proinflammatory gene expression promoted by the CNIs (Fig. 7C,D). Interestingly, pharmacological TLR4 inhibition also prevented CNI upregulation of ET-1 mRNA expression, a key factor involved in the endothelial dysfunction (Fig. 7D). To reinforce these results, studies were done in aortas from TLR4^−/−^ mice. CsA or tacrolimus stimulation did not upregulate CCL2, IL6, ICAM-1 or ET-1 gene expression in isolated aortas from TLR4^−/−^ mice, thus confirming that TLR4 is a key intermediary of pathological vascular responses to CNI. Importantly, TLR4 deficient aortas were still responding to the inflammatory cytokine TNF-α, a not TLR4 ligand, as revealed by the increased CCL2, IL-6, ICAM-1 and ET-1 mRNA levels (Fig. 7E).

VSMCs play a central role in vascular inflammation and injury and may be a CNI target. Thus, we explored whether VSMCs contribute to the CNI-induced aortic inflammation. As in the case of endothelial cells and aorta, VSMCs increased CCL2, IL-6 and TNF-α mRNA levels in response to CNIs and this was prevented by TLR4 inhibition (Fig. 7F). This result suggests that activation of the TLR4 pathway by CNIs is highly conserved in the most relevant cell types of the vascular wall.

### CNI-produced inflammation is linked to TLR4-dependent ROS production

**Figure 8:**
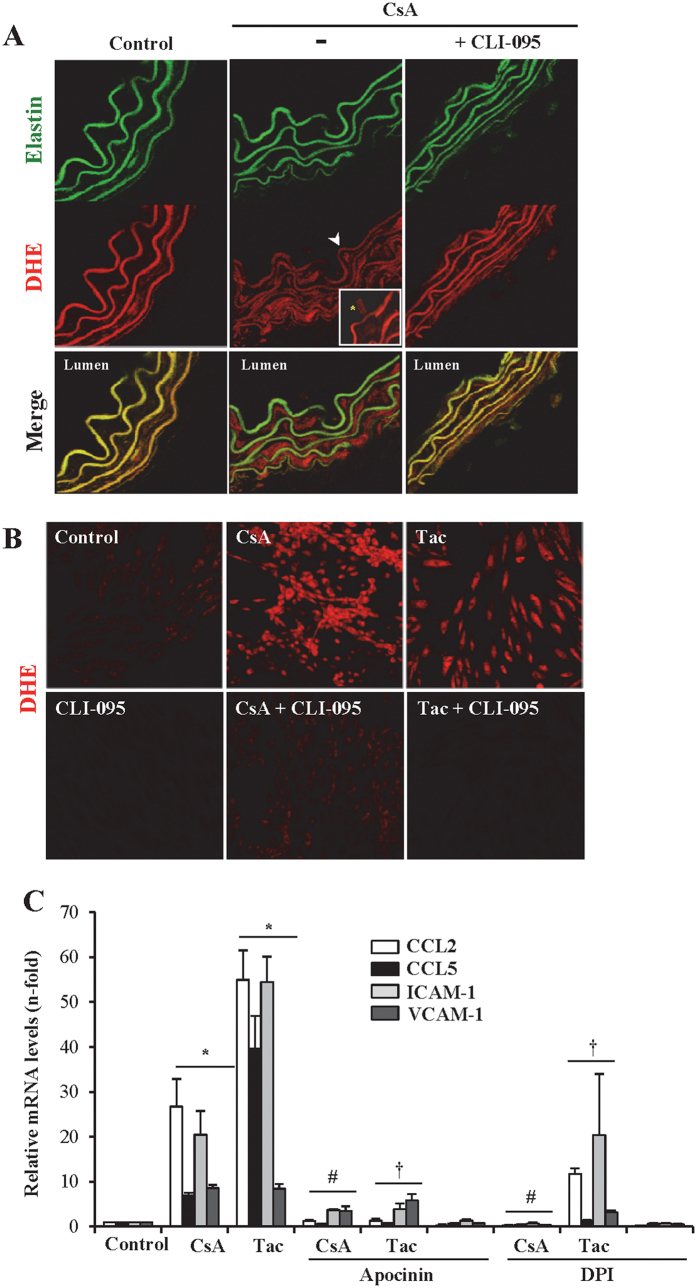
Activation of the TLR4 pathway by CsA and tacrolimus increases the production of O_2_^−^/ROS as inflammation mediator in aorta and endothelial cells. (**A**) Confocal photomicrographs of aortic tissue sections treated with 10 μg/ml CsA for 6 h showing an increased O_2_^−^/ROS production measured as DHE staining (red fluorescence). Elastin auotofluorescence (green) shows the structure of the artery that contrast with the red fluourescence indicating that O_2_^−^/ROS was mainly located in VSMCs (media layer) and also in endothelial cells (intima layer). White arrowhead shows DHE positive VSMCs and asterisk point DHE positive endothelial cells, which are showed at a larger augment (inset). Aortas subjected to pharmacological TLR4 inhibition with CLI-095 show a lesser DHE staining. Original magnification x630. (**B**) Representative confocal photomicrographs of O_2_^−^/ROS production measured as DHE positive staining in murine endothelial MS1 cells treated with 10 μg/ml CsA (A) or 20 μg/ml Tac for 6 h. Cells subjected to pretreatment with CLI-095 before CsA or Tac stimulation exhibit a basal DHE staining after treatment with CsA or Tac. (**D**) Gene expression of proinflammatory factors and adhesion molecules (CCL2, CCL5, VCAM-1 and ICAM-1) was assessed by PCR in MS1 cells treated with 10 μg/ml CsA (**A**) or 20 μg/ml Tac for 6 h, both in the presence or in the absence of the NADPH inhibitors Apocinin (10 mM) and DPI (10 μM) added 1 h before the stimuli. The bar chart represent the Media ± SEM of a set of three independent experiments. *p ≤ 0.05 vs control; ^#^p ≤ 0.05 and ^†^p ≤ 0.05 vs CsA or Tac alone, respectively.

Oxidative stress is a key mediator of inflammation in vascular disease so we explored whether CNIs increased reactive oxygen species (ROS) production in *ex vivo* cultured aortas exposed to CNIs alone or following exposure to CLI-095. Compared to untreated control aortas, those exposed to CsA showed an increased O_2_^−^/ROS production, mainly located to VSMCs but also detectable in endothelial cells (Fig. 8A). By contrast, pre-treatment with CLI-095 before CsA addition prevented ROS production as assessed by DHE staining, proving that TLR4 activation regulates O_2_^−^/ROS production (Fig. 8A). Similar results were obtained when aortas were incubated with tacrolimus (data not shown).

The precise contribution of TLR4-dependent O_2_^−^/ROS increases to CNI-induced vascular inflammation was further evaluated in endothelial cells chosen as model of CNI-induced TLR4 signaling. Similar to aorta experiments, pharmacological TLR4 inhibition also repressed O_2_^−^/ROS production as assessed by DHE staining (Fig. 8B). Further, the NADPH inhibitors apocinin and DPI prevented the the upregulated CCL2, CCL5, VCAM-1 and ICAM-1 mRNA expression induced by CsA and tacrolimus (Fig. 8C). Altogether, these results point to oxidative stress and O_2_^−^/ROS production as a contributor to CNI-induced vascular inflammation and localized O_2_^−^/ROS formation downstream of TLR4 activation.

## Discussion

We have shown that CNIs modulate vascular behaviour by inducing proinflammatory events, namely endothelial activation as well as production of inflammatory cytokines, through TLR4 activation and ROS generation in endothelial cells and VSMC as well as in aortic tissue.

The role of the innate immunity and associated inflammatory mechanisms in the development of the vascular adverse effects of CNIs had not been specifically addressed previously. Vascular inflammation is a central process underlying endothelial dysfunction, vascular damage and progression of cardiovascular disease^28^,^29^. Recent studies from our group disclosed that CNIs promote tubular inflammation in kidneys^10^. The present research demonstrated that CNIs also have proinflammatory effects on endothelial cells and VSMC, thus widening the spectrum of cell types susceptible to the inflammatory action of CNIs. A prior report explored the modulation by CsA of TNF-α/LPS-induced endothelial cell inflammation and observed that CsA promoted inflammation-induced monocyte adhesion to human intestinal endothelial cells but paradoxically, reduced inflammation-induced iNOS activation and adhesion molecule expression^30^. Our results demonstrating that CNIs by themselves increased the expression of a complete spectrum of endothelial activation markers and inflammation mediators in cultured endothelial cells and murine aortas in a TLR4-dependent manner provide a better understanding of CNI-induced vascular injury and provide a knowledge frame to explain prior observations. Thus, direct induction of endothelial activation and cytokine production by CNIs may contribute to allograft vascular injury as well as to nephrotoxicity. While endothelial injury and inflammation negatively impact on normal VSMCs function, an inflammatory response from VSMC may also contribute to endothelial injury and dysfunction. Results obtained in isolated aortas and cultured VSMC point to a potential role of aorta-generated inflammatory cytokine, such as IL-6 and TNF-α, secreted in response to CNIs, in an autocrine or paracrine loop resulting in additional endothelial activation, cytokine production and worsening of the systemic vascular function^13^,^29^.

NF-κB is the major mediator of the inflammatory response in vascular cells and other cell types, and as such, it is an attractive target for chemoprevention of inflammatory diseases^22^,^31^. NF-κB expression correlates with upregulation of chemokines in human cardiovascular disease and in experimental models of vascular damage^22^,^28^,^32^,^33^,^34^. We previously demonstrated that CNIs activate NF-κB in renal tubular cells *in vitro* and in a mouse model of CsA nephrotoxicity^10^. In the present work, by means of pharmacological inhibition of IκB kinase and IκBα stabilization with parthenolide, we showed that NF-κB mediates the CNI-induced synthesis of key proinflammatory cytokines and adhesion molecules in endothelium. Moreover, CNIs activated NF-κB in the entire vascular wall, including VSMC. This is relevant for CNI toxicity since NF-κB inhibition with parthenolide prevents experimental atherosclerosis and renal damage^33^,^35^,^36^. Thus, synthetic or naturally occurring NF-κB small molecule inhibitors should be explored to prevent CNI-induced vascular inflammation.

Increased activity or expression of TLRs, particularly TLR4, has been associated with vascular inflammation^19^,^37^,^38^. In accordance with previous observations from our group on CNI-induced TLR activation in tubular cells, MyD88 silencing in endothelial cells prevented the induction of proinflammatory and endothelial activation markers by CsA and tacrolimus. MyD88 is an adaptor protein that participates in the formation of proximal complexes with TLR2 and TLR4^12^,^39^. However, in our experiments, cultured endothelial cells did not respond to the TLR2 agonist lipoteichoic acid (data not shown). This allowed focusing research on the role of the TLR4 pathway in the proinflammatory effects of CNIs. Both pharmacological and genetic targeting of TLR4 confirmed its role in CNI-induced inflammatory responses in endothelial and vascular cells. Remarkably, TLR4 was also required for CNI-induced ET-1 gene expression. ET-1 is a potent vasoconstrictor peptide involved in hypertension^40^. This result reinforces the idea of endothelial inflammation, activation and dysfunction being related phenomena in response to CNIs and disclosed a central role of TLR4 as a gatekeeper controlling all of these responses in vascular cells.

We also identified oxidative stress as a key event downstream of TLR4 in aortic endothelial and vascular cells exposed to CNIs. Oxidative stress contributed to the CNI-induced synthesis of proinflammatory mediators and endothelial stress markers. A pathway implying a TLR4-dependent ROS formation, NF-κB activation and inflammation was reported in VSMCs subjected to AngII treatment^18^,^23^. CsA is a well-known inducer of oxidative stress and cell damage in the vasculature, and notably, in renal tissue^41^,^42^ Likewise, previous work from our laboratory showed that ROS mediate CsA-induced cell death in endothelial cells through activation of the JAK2/STAT3 pathway^43^. Moreover, it is well known that JNK is activated by oxidative stress^44^. In endothelial MS1 cells, both JAK2 and JNK are activated by CNIs and their inhibition results in the blockade of the CNI-provoked proinflammatory responses (data not shown). Downstream transcription factors of these protein kinases, namely STAT3 and AP-1, an even their activating protein kinases, interact with NF-κB to activate inflammatory responses^45^,^46^,^47^. Thus, our present results strongly suggest a role of redox imbalance and ROS generation in the TLR4-dependent signaling and NF-κB-dependent inflammation.

The observation that vascular and kidney toxicity profiles of CsA and tacrolimus are similar in experimental models and humans, suggests that their interaction with calcineurin could be a common element involved in the CNIs adverse effects. In endothelial cells, the interaction of CNIs with calcineurin is required for decreasing eNOS activity and NO content. However, calcineurin was dispensable to increase TGFβ receptor expression and arteriolar hyalinosis or to potentiate the LPS-induced leukocyte binding to endothelium, as suggested by similar results obtained with rapamycin^9^,^30^,^48^. In macrophages, unlike in T cells, calcineurin hindered NF-κB and IRF activation by inhibiting TLR-mediated signaling through MyD88 and TRIF, and calcineurin targeting by genetic means or by CNIs resulted in increased synthesis of proinflammatory cytokines^49^. In the present manuscript, unlike in macrophages, calcineurin deficiency did not increase proinflammatory factor secretion. By contrast, it partially inhibited but did not impede the overall CNI-induced proinflammatory response, suggesting that binding of CNIs to calcineurin may enhance the proinflammatory effect, but is not an absolute requirement. In accordance with this result, calcineurin deficiency mildly reduced evidence of NF-κB activation at the IκBα phosphorylation level, but this fact did not result in modification of p65 phosphorylation/activation. This observation is in agreement with the reported phosphorylation of p65 at Ser536 being independent of IκBα^50^. Moreover, according to the proposed role of Ser536 p65 phosphorylation promoting inflammatory gene expression^51^, maintenance of Ser536 p65 phosphorylation was associated to a largely preserved inflammatory gene expression following CNI exposure. By contrast, our results revealed that calcineurin deficiency abolished the CNI-induced TRIF signaling. The TRIF pathway promotes sustained NF-κB-dependent responses following TLR4/MyD88 signaling^52^. In this manner, we could envisioned a role of calcineurin suppressing long lasting activation of TLR4 and the loss of this inhibitory feedback as a possible mechanism contributing to exacerbation of CNI toxicity. Overall, this set of results show that calcineurin availability is not an absolute requirement, but it may contribute to CNI-induced proinflammatory events in endothelial cells. Thus, interaction with calcineurin could stabilize CNIs inside cells and increase toxicity.

In conclusion, we have shown that TLR4 mediates endothelial inflammation, activation and dysfunction induced by CNIs. These effects appear to be dependent on the TLR4/MyD88/NF-κB and TLR4/TRIF/NF-κB pathways and involved oxidative stress generation downstream of TLR4 and upstream of NF-κB activation. Although calcineurin expression appeared to heighten CNI-mediated TLR4 signaling and hence NF-κB-dependent cytokine and adhesion protein synthesis to some extent, this final proinflammatory effect was largely observed even in calcineurin-deficient cells. By contrast, results showed that TLR4 is an essential mediator of CNI proinflammatory effects in the vasculature. The association between TLR4 inactivation and improving of cardiovascular disease is becoming increasingly clear from a range of animal models dealing with this topic^17^,^18^,^53^,^54^. Indeed, TLR4 blockade arises as potential therapy against many manifestations of transplant and CNI-associated vascular disease, including inflammation, hypertension and atherosclerosis. CNI immunosuppression is presently unavoidable to overcome allograft rejection, although prevention of CNI toxic side effects, including vascular toxicity, is yet an unmet necessity. Adjuvant therapies could be an acceptable strategy to improve immunosuppression based on CNIs. In such a way, TLR4 inhibition could be envisioned as a potential therapeutic approach for prevention of CNI-associated vascular toxicity after transplant.

## Additional Information

**How to cite this article**: Rodrigues-Diez, R. *et al*. Calcineurin inhibitors cyclosporine A and tacrolimus induce vascular inflammation and endothelial activation through TLR4 signaling. *Sci. Rep.* **6**, 27915; doi: 10.1038/srep27915 (2016).
